# Co-design personal sleep health technology for and with university students

**DOI:** 10.3389/fdgth.2024.1371808

**Published:** 2024-04-09

**Authors:** Zilu Liang, Edward Melcer, Kingkarn Khotchasing, Nhung Huyen Hoang

**Affiliations:** ^1^ Ubiquitous and Personal Computing Lab, Faculty of Engineering, Kyoto University of Advanced Science (KUAS), Kyoto, Japan; ^2^ Alternative Learning Technologies and Games Lab, Department of Computational Media, University of California, Santa Cruz (UCSC), CA, United States

**Keywords:** mHealth, sleep health, co-design, participatory design, sleep tracking, personal health informatics

## Abstract

University students often experience sleep disturbances and disorders. Personal digital technologies present a great opportunity for sleep health promotion targeting this population. However, studies that engage university students in designing and implementing digital sleep health technologies are scarce. This study sought to understand how we could build digital sleep health technologies that meet the needs of university students through a co-design process. We conducted three co-design workshops with 51 university students to identify design opportunities and to generate features for sleep health apps through workshop activities. The generated ideas were organized using the stage-based model of self-tracking so that our findings could be well-situated within the context of personal health informatics. Our findings contribute new design opportunities for sleep health technologies targeting university students along the dimensions of sleep environment optimization, online community, gamification, generative AI, materializing sleep with learning, and personalization.

## 1 Introduction

**Figure 1:**
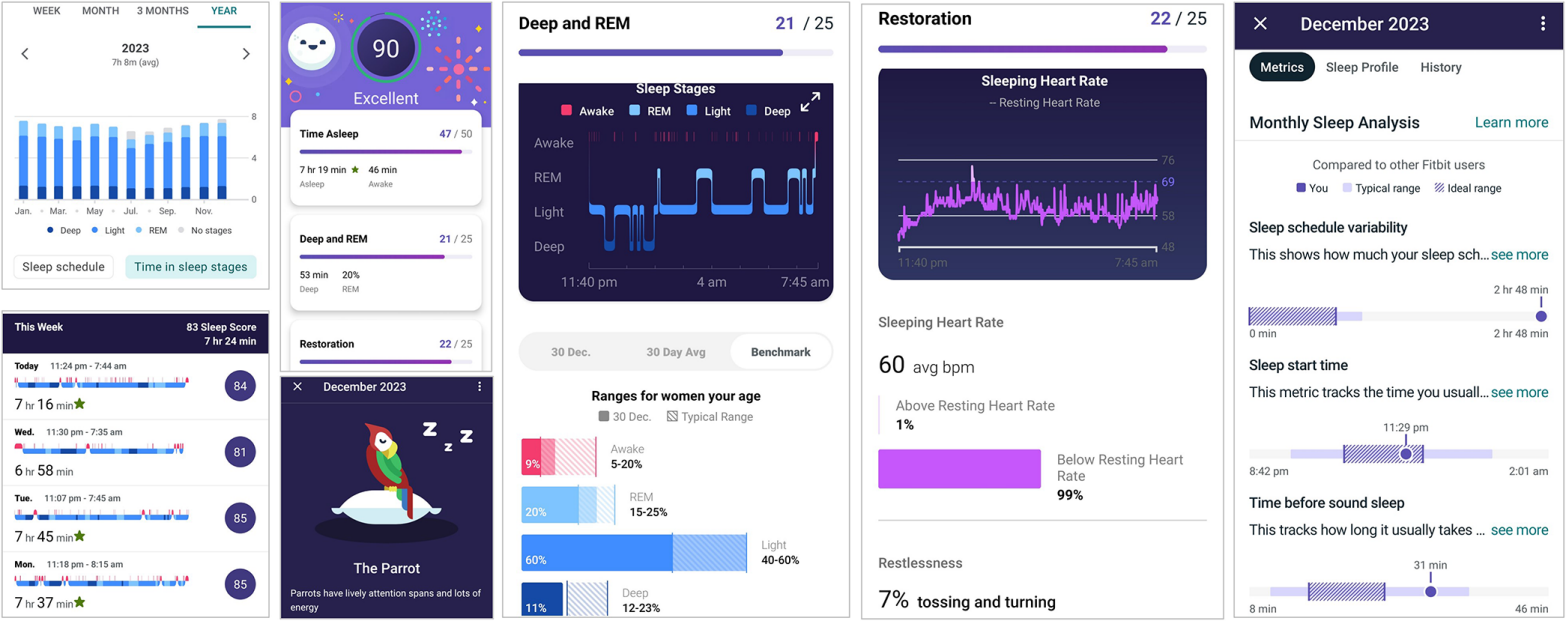
Sleep profile features of Fitbit app. The sleep data include the daily, weekly, monthly, and yearly summary of sleep quality, sleep stages, sleep regularity, and heart rate during sleep. The premium Fitbit Sleep Animal feature assigns an animal that mirrors a user’s sleeping style in the past one month. For example, parrot-type sleepers tend to keep a consistent sleep schedule and usually get sufficient sleep each night. They have good amount of deep sleep but can be light on REM sleep.

University students often experience sleep disturbances and disorders. There is a large body of sleep science research investigating the mechanism and causes underlying the sleep problems of university students and their associated consequences (8, 2, 5, 4, 6, 3, 7, 1). Sleep problems particularly relevant to this population include having difficulty initiating sleep, maintaining sleep, and waking up in the morning. Digital health technologies such as smartwatches and mobile apps, despite having the risk of causing sleep disruption, could be leveraged to deliver just-in-time sleep interventions if designed carefully (9). As most university students are technology savvy and spend significant time interacting with their smartphones, digital sleep health technologies present a great opportunity for sleep health promotion targeting this population. These technologies could be used to self-track a flux of data in a naturalistic setting: not just sleep data per se, but also daily activities, physiological signals, and biometrics. There is also increasing evidence that consumer sleep trackers are comparable to medical devices in detecting bed time and wake up time as well as the total sleep duration, but their accuracy in measuring sleep stages requires further improvement (10, 11). In an adjacent area, digital health researchers have intensively studied the potential of ubiquitous sleep-tracking technologies in promoting sleep health in the public. Off-the-shelf consumer sleep health technologies mostly support sleep monitoring and tracking in daily life settings (e.g., Figure 1), but leave it up to users to interpret the sleep data within the context of their lives (12). Many studies have attempted to fill the gap by developing tools that allow users to discover the interrelationships between sleep and their lifestyles by making sense of a flux of personal data (14, 13). A few studies further investigated the potential of self-experimentation for improving sleep (15). Prior studies have examined the usability, credibility and validity of sleep health technologies for adults (12, 13, 11, 17, 16), high school students (14), and young children (18).

However, research that engages university students in designing and implementing digital sleep health technologies is lacking. University students experience a natural change in circadian rhythm (19, 20). Many students suffer from sleep phase delay, with the most common symptoms being going to bed late and having difficulty waking up in the morning. On top of that, unhealthy lifestyle and mental health conditions are also known to have reciprocal relationships to poor sleep quality (8, 24, 21, 3, 22, 23). The COVID-19 pandemic further exacerbated the prevalence of sleep disorders among university students (25). Sleep science and public health research have generated a set of coping strategies, ranging from sleep literacy education (e.g., disseminating knowledge of healthy sleep habits and healthy lifestyle) (3) to adjusting university policies and class schedules that encourage healthy and adequate sleep (27, 26). However, how to implement those strategies remains open for further investigation. To date, only a few studies have focused on creating sleep health interventions for university students (30, 28, 29). Furthermore, existing interventions are either not centered on the needs of university students or not delivered through digital media, which may limit their efficacy and accessibility to this population.

This study set out to fill the gap by exploring the design opportunities surrounding digital sleep health technologies for university students. Notably, we sought to understand how we could build digital tools that meet the needs of this user segment through a co-design process. Co-design stems from participatory design, where the people who will use a system end up playing a critical role in designing it (31). For co-design processes, stakeholders are treated as equal collaborators or can even take the lead in the design process rather than having limited design contribution roles (32). In this way, co-design shifts responsibility and control so that “clients” or users of services become active partners in designing and shaping those services, rather than being passive recipients (33). Co-design has attracted attention in the healthcare domain (34), and has been widely applied to the design of mHealth technologies for disease self-management (35). In the context of sleep health, co-design was recently applied to co-create sleep management guidelines (36).

To the best of our knowledge, our study is the first to apply the co-design approach to the creation of sleep health technologies for university students. More specifically, we conducted three co-design workshops with target users to generate design ideas through a series of workshop activities. Participants were guided to reflect on the factors that cause sleep disruption, the limitations of existing sleep technologies, and to generate design ideas that promote healthy sleep habits and improve sleep. We frame our findings using the stage-based model of self-tracking (37) within the context of personal health informatics, and contribute new design opportunities for sleep health technology targeting university students.

## 2 Method

### 2.1 Participants

We adopted a co-design approach that involves a cohort of university students in designing sleep health technologies to address sleep problems and meet the needs of this population more broadly. We distributed flyers around the campus of Kyoto University of Advanced Science (KUAS) to recruit workshop participants. The inclusion criteria were being fluent in English and being enrolled in a university at the time when the workshops were conducted. This study was approved by the Ethics Review Board of KUAS. All participants signed a written informed consent form. Each participant received an Amazon gift card worth approximately US$20 as compensation for their time.

### 2.2 Co-design workshops

We conducted three co-design workshops in the summer and autumn of 2023. The first two authors conceptualised and designed the workshop content. The second author facilitated the first two workshops, and the first and the third author facilitated the last workshop. The three workshops adopted the same content flow, each lasting for 3 h. The first activity was an ice-breaking session where everyone introduced themselves. The facilitator(s) then provided a short presentation of the workshop goal and some background information. After that, the participants split into multiple groups of 4-6 people and went through a series of activities. Notably, the empathy map activity (38) prompted the participants to reflect on the factors that make it hard to sleep, their feelings when they experience sleep disruption, and their opinions of current sleep health technologies. The brainwriting activity (39, 40) asked the participants to write up app ideas that could possibly engage users over a long period of time.

### 2.3 Data analysis

**Figure 2:**
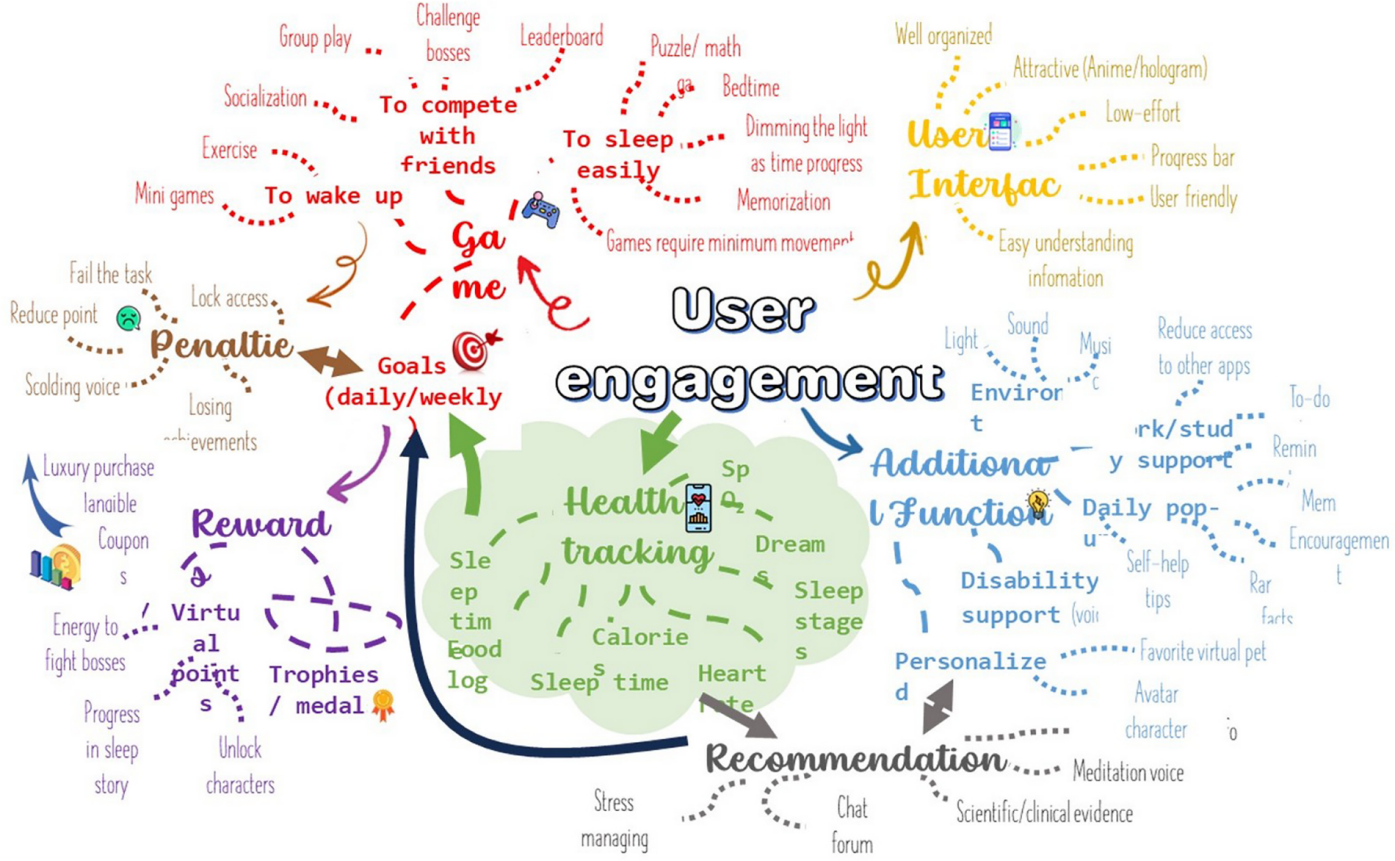
A thematic map created by one of the authors.

A variety of data was generated in the workshops. Our analysis centres on the empathy map and the brainwriting activities because, for the purpose of this paper, we are mostly interested in identifying design opportunities for computing technologies to support the sleep health of university students and the features that could potentially engage them over a long period of time to achieve sustained healthy behaviour change. For data generated using the empathy map, we re-grouped the sticky notes using the affinity diagram method (41). For the design ideas generated during the brainwriting sessions, we imported them into the Dovetail software and performed thematic analysis following the process outlined in Braun and Clarke (42). Three authors independently coded the data and created a thematic map. An example of the thematic map is shown in Figure 2. We discussed the codes and themes together to resolve any conflicts. Guided by the stage-based model of personal informatics systems (37), we then mapped the themes to each of the four stages of self-tracking, i.e., preparation, collection, reflection, and action.

## 3 Results

In total 51 participants (6 female) aged between 18 and 35 years old attended the workshops, 19, 11, and 21 for each workshop, respectively. All students are enrolled in the Faculty of Engineering at KUAS. Fourteen participants (27.4%) had prior experience using a smartwatch or wristband for sleep tracking, while none of the students had previously participated in a co-design process.

### 3.1 Sleep disturbance factors

Many factors surfaced as participants brainstormed about factors that cause sleep disruption. Those factors are grouped into four categories: poor daily habits, negative emotions, workload of study and part-time job, and poor sleep environment.

Participants primarily attributed their sleep problems to bad daily habits. Digital device usage is the most frequently mentioned sleep disruption factor under this category. Approximately two-thirds of the participants acknowledged that they often have too much screen time at night on social media (TikTok, Instagram, Facebook), streaming service platforms (YouTube, Netflix) or online shopping websites. Late dinner or late-night snacking is the second most frequently mentioned sleep disruption factor. Other factors include going to bed hungry, stimulant (caffeine, alcohol, tobacco) use, irregular sleep schedule, taking naps late in the afternoon, and late-night social activities. Negative emotions are also frequently mentioned as the reason for sleep problems. Almost half of the participants mentioned that overthinking (e.g., “*the environment is too quiet; I need some distraction from my self-thoughts*”) and worries about exams, relationships and the future make it hard to fall asleep. New university students often mentioned the adaptation stress from living in a new environment, learning a new language, and embracing a new culture. Some participants experienced anxiety and mood swings, stating that “*feeling homesick and lonely makes it hard to sleep*”. Participants considered the workload of study, research or part-time jobs to be significant contributors to interrupted sleep schedules. Approximately half of the participants mentioned that they had to stay up late doing assignments or research to meet deadlines, and they confessed that it may not be necessarily due to a heavy workload but rather a consequence of poor time management. On the other hand, some participants just like to stay up late because they sometimes have “*random eureka moments*” at night or they are more productive (e.g., “*better inspiration for writing*”), or simply because they wanted to do more things such as working on new side-projects. Last but not least, a non-ideal sleep environment, such as noise from the neighbourhood, bright ambient light, and uncomfortable temperature, could contribute to sleep loss.

It is surprising that participants do not always associate sleep disruption with negative consequences. While stress, anxiety and frustration are often induced when people cannot sleep, some participants contrarily felt “*happy because I can do my assignment*”. Some mentioned that they are more creative and “*get the best ideas*” when they cannot sleep. Others mentioned that they sometimes were more self-conscious and thoughtful late night, materializing insomnia as an opportunity to reflect on life.

### 3.2 Opinions on current sleep health technologies

Participants’ opinions were divided as to whether they would like to use existing sleep health technologies. On the positive side, these technologies are considered potentially useful in bench-marking one’s sleep quality and sleep habits and in creating a good user experience. On the flip side, participants listed several barriers to technology adoption, including usability issues, potential adverse effects, limited usefulness, technology immaturity, and privacy concerns.

The major reason for participants to endorse existing sleep health technologies seems to be the perceived usefulness. They would like to use sleep apps to monitor the status quo of their sleep quality and sleep stages (notably deep sleep), as well as “*comparing to the rest of the population*”. One participant considered these technologies as a tool for reflecting on his sleep habits. Participants also expect sleep apps to help them develop good sleep hygiene, especially in terms of nighttime sleep routines and sleep schedules. Only a few participants expected sleep technologies to actually help them sleep better or to have a positive impact on their general health. Meanwhile, approximately one-fifth of the participants would like to use sleep technologies “*just out of curiosity*” or “*for a new experience*”.

The reasons for not using sleep health technologies are diverse. First, more than one-third of the participants mentioned usability issues of sleep gadgets or apps. Some are not comfortable with having to wear a smartwatch during sleep, and others are not willing for a sleep app to consume the resources of their smartphones, stating that it will “*take up the storage*” or “*drain the battery*”. Second, participants are worried about potential adverse effects of using these technologies, such as being distracted from sleep, causing rumination, interrupting sleep due to blue light from the smartphone screen, becoming “*dependent on technology*”, and the “*feeling of being judged*”. In addition, some participants questioned the usefulness of technologies. They doubted that the sleep data would lead to actionable insights that eventually fix sleep problems. Three participants did not think they need such technology either because they “*sleep well most of the time*”, or they trust their own feelings of sleep, or they get the best ideas when they cannot sleep. A few participants were skeptical about the accuracy and validity of sleep technologies, and pointed out their lack of trust in large technology companies.

### 3.3 Design ideas for future sleep health technologies

**Figure 3:**
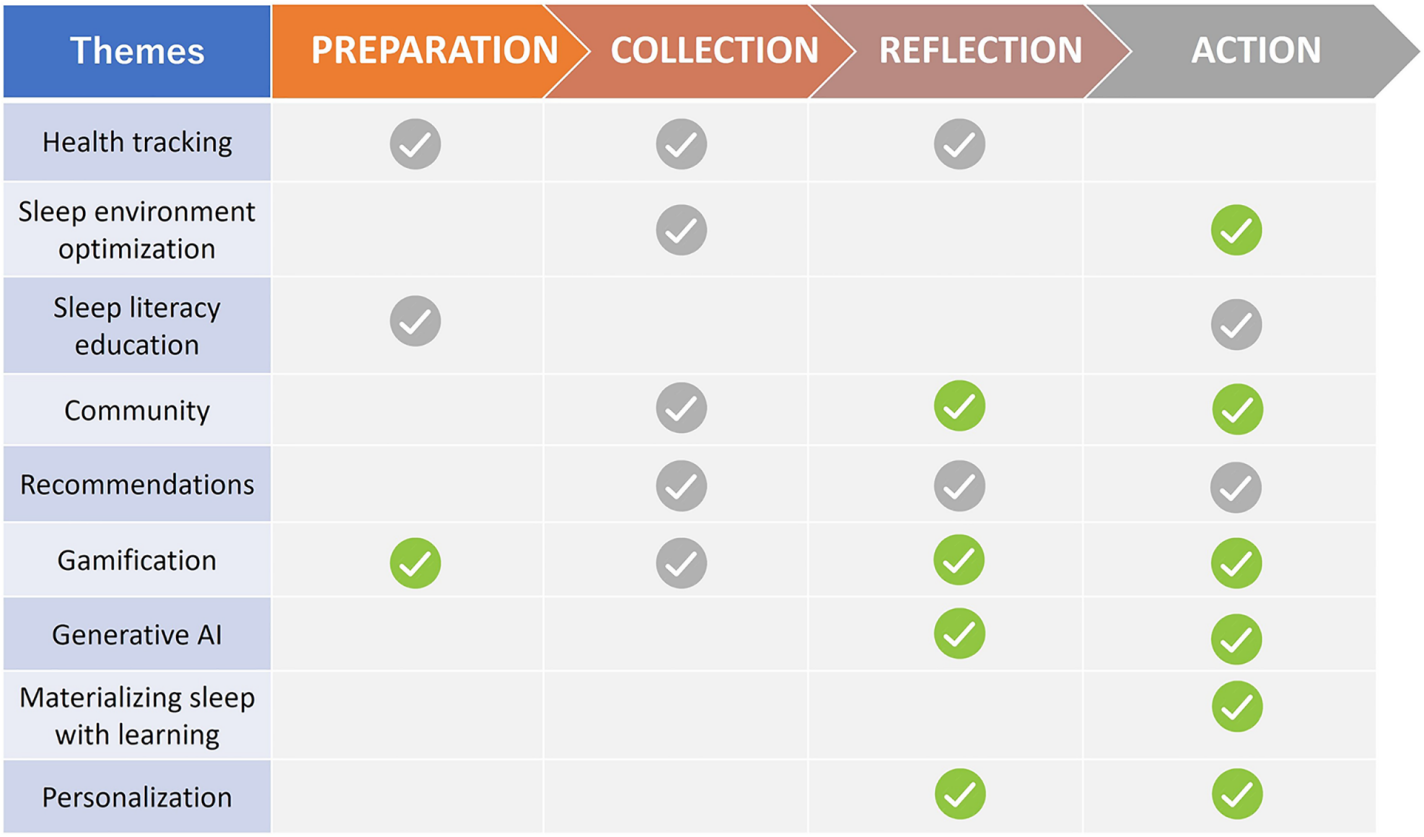
Mapping the themes surfaced in the brainwriting activity to the stage-based self-tracking model. Green check marks indicate new design ideas generated in the workshops, and grey check marks indicate ideas that overlap with existing features of sleep health technologies.

**Figure 4:**
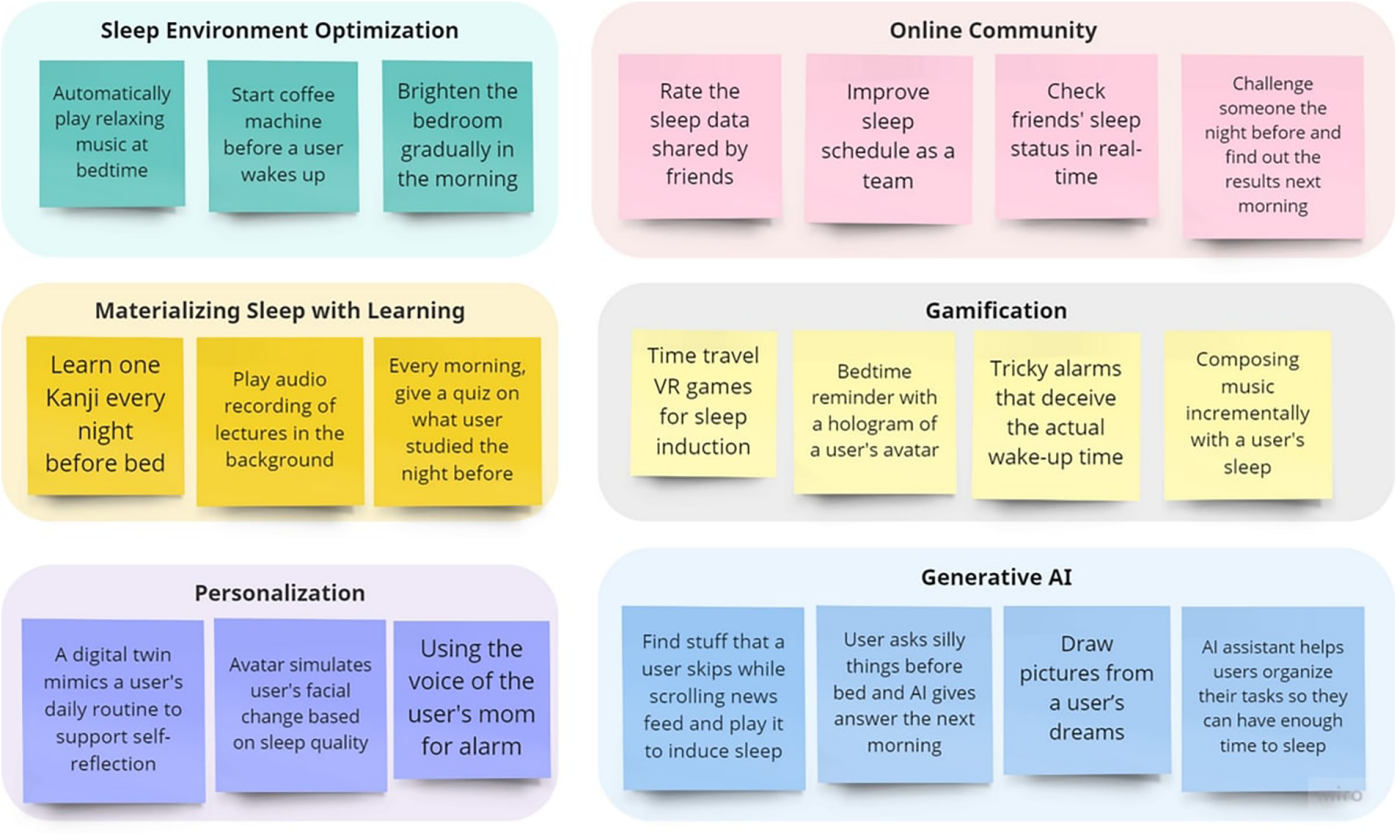
Example design ideas that were generated under each theme. Many of these ideas are novel and could be useful for developing sleep health technologies for university students.

Thematic analysis of the brainwriting data generated nine themes. Figure 3 shows a high-level overview of the findings with the themes mapped to each self-tracking stage. Three themes—health tracking, sleep literacy education and recommendations—largely overlap with prior findings in personal sleep-tracking (43, 17, 13) and thus will not be discussed further in this section. In what follows, we present the remaining six themes in detail. Example design ideas under each of the six themes are illustrated in Figure 4.

#### 3.3.1 Sleep environment optimization

Sleep environment is known to affect sleep quality. This is supported by the results generated from the empathy map, where participants mentioned a non-ideal sleep environment as a major sleep disruption factor. Keeping an ideal sleep environment that is quiet and dark, not too cold or too hot, is an important aspect of good sleep hygiene (44). Systems and devices that allow users to monitor bedroom environments have been previously developed (45). However, our participants went one step further to highlight that sleep health technologies should not only support the monitoring but also the setup of an ideal sleep environment. Participants envisioned the Internet of Things (IoT) and smart home technologies that automatically control the ambient light, temperature, humidity, and background noise for sleep induction. While the concept of automatic setup of a sleep environment before bed has been recently explored in a few smart home studies, our participants posited that intelligent systems could orchestrate the sleep environment to users’ sleep state throughout the night and help users wake up in the morning (e.g., “*brightening the bedroom gradually as the morning approaches*”). Since participants considered the blue light of electronic devices as a major harmful factor to sleep, they proposed to couple the sleep environment optimization with no disturbance or blue light blockage on users’ smartphones, such as the Sleep Focus feature of iPhone or the Bedtime mode of Android phones. Moreover, several interesting IoT applications were proposed to facilitate the establishment of a regular sleep-wake cycle. For example, an IoT system could be developed to “*automatically play boring stories and relaxing music at bedtime*“ to help users fall asleep, and in the morning, it could “*start coffee machine*” and “*adjust bed position*” to wake up users.

#### 3.3.2 Online community

The collaborative nature of self-tracking practice has been explored in prior studies (47, 46). Supporting collaborations by establishing online communities has become a popular feature of self-tracking technologies (48). In the same vein, prior sleep tracking research positioned sleep as an activity within users’ social context. Several studies have investigated the collaborative aspect of sleep tracking, notably in the domains of online communities (49), families (51, 50), long-distance relationships (52), and workplace (53).

While existing community features heavily centre on data and experience sharing in online forums or connecting users to medical professionals (54), our participants envisioned more activities for multi-user scenarios. Some of these features include allowing users to “*rate the sleep data shared by friends*” and “*improving sleep schedules as a team*”. These activities would lead to sustained engagement of multiple users either synchronously or asynchronously, supporting their reflection on sleep and action-taking in a collaborative fashion.

#### 3.3.3 Gamification

Gamification is defined as the incorporation of game elements into non-game contexts (55), and was a dimension highly appreciated by our participants. There are a variety of different gamification techniques (56). However, prior studies have mainly applied gamification features such as rewards, level-up, and story process/narrative to engage users at the data collection and reflection stages (57). Our participants proposed a variety of design ideas that leverage game elements to engage users at the other two stages, i.e., preparation and action, of a self-tracking cycle. For example, users could be prompted to configure the game setting (e.g., “*allow users to customize their avatars or game narratives*”) and set goals they intend to accomplish in the game when they plan for the sleep self-tracking cycle. Games that wake up users with cognitive or kinetic tasks may facilitate a smoother transition from sleep to wakefulness in the morning. Several specific design ideas under this theme are listed below.

• Virtual reality (VR) games that provide an immersive gaming experience (e.g., “*time travel*” or “*starring sky*” for sleep induction, “*replicate dreams with VR*”, “*bedtime reminder using the hologram of a user’s avatar*”.
• Virtual goods that reward users for their compliance to good sleep hygiene and that can be traded with other users.
• Tricky alarms that deceive the actual wake-up time (e.g., “*let the alarm ring at 7:00 am but show 10:00 am on the clock face*”).
• Punishments/penalties that discourage unhealthy sleep habits (e.g., “*deleting users’ profile photos if they stay up late*”, “*cash penalty if users do not follow their pre-set sleep schedule*”).

#### 3.3.4 Generative AI

The most unique ideas generated in the co-design workshops are those related to generative AI, or ChatGPT in particular. Those ideas were mapped to the reflection and action stages of the self-tracking cycle. Participants proposed that ChatGPT may be employed to induce sleep at bedtime by generating boring texts, audio, or random keywords relevant to users. One specific idea is to “*find stuff that a user skips while scrolling news feed and play it to induce sleep*”. Some other AI features that serve to facilitate the transition from sleep to wakefulness include “*play YouTube videos that relate to things a user likes*” or “*generate and display positive quotes or funny memes to wake up users*”. Since poor time management often compounds the sleep disruption effect of deadlines, some participants devised the solution of an AI assistant powered by ChatGPT, which helps users smartly manage their schedules and organize their tasks so that they could have sufficient sleep at night. GhatGPT may even have the potential to support reflection, e.g., by drawing pictures from users’ dreams.

#### 3.3.5 Materializing sleep with learning

We found that a major difference between university students and the general adult population is that students always place a priority on their studies. Several participants mentioned that features that could benefit their study and learning are likely to keep them engaged with the technology over a more extended period of time. Interesting features were proposed to couple sleep with study, e.g., “*learn one Kanji every night before bed with a mnemonic*”, “*play audio recordings of lectures in the background for the preparation of tomorrow’s lectures*”, “*every morning give a quick quiz on what a user studied the previous night*”, and “*motivate users to wake up by displaying the to-do list of the day*”.

#### 3.3.6 Personalization

Participants consider personalization as a potential direction for engaging users in long-term behavioral change. A distinctive idea is based on the concept of a digital twin (58, 60, 59), or a digital representation of a user in the virtual space. Some participants proposed using digital twin as a user’s alternative reality to replicate the user’s sleep behavior (e.g., “*a virtual character mimicking the daily routine of a user to support self-reflection*”) and to simulate the consequences of users’ poor sleep hygiene (e.g., “*avatar simulating your appearance change based on how much sleep users routinely get*”). Interactions between a user and its digital twin may be designed to facilitate sleep education as well, e.g., “*message from users’ avatar to educate them how to sleep better*”. Other personalized features proposed by the participants include “*generating stories based on a user’s past memories*” and “*using the voice of the user’s mom for alarms or bedtime reminders*”.

## 4 Discussions

Analysis of the empathy map identified several sleep disruption factors, including screen time, emotional and academic stress, anxiety, irregular sleep schedules, eating habits, and uncomfortable sleep environments due to noise and light. Most of these factors overlap with findings from prior sleep health studies on university students and adolescents (8, 24, 61, 21, 3, 22, 23). While lacking physical activity and a sedentary lifestyle is widely considered as a negative factor to sleep (14, 24, 13), it was not mentioned by any participants in the workshop. Hoping to understand their sleep status quo is the main value proposition the participants imposed on existing sleep health technologies, while several usability issues, potential adverse effects, limited usefulness, and low accuracy and credibility are the main barriers for participants to adopt those technologies. These findings echo prior studies on sleep health technology for the general population (17, 12).

One surprising finding from the empathy map is that having a problem falling asleep or staying asleep is not always considered as something negative or something that should be completely avoided at all costs. Staying up late is sometimes not driven by deadlines or passive procrastination but rather by choice or even preference. This is probably due to age-related physiological change, which results in a delayed circadian phase in this population (19, 20). Our finding shows that students not only think of sleep problems as a normal part of student life, which echoes a recent study (30), but also sometimes prefer sacrificing sleep for something else. Some participants felt more productive and creative late at night. For them, sleep disruption is transformed into a much-needed opportunity to accomplish more or to reflect on life. This new finding would not have been made possible without using the co-design approach that values the voice of the target users.

Many design ideas surfaced through the brainwriting activity in the co-design workshops. Some of the ideas directly target the sleep disruption factors and the limitations of existing sleep technologies surfaced from the empathy map, such as automated sleep environment optimization, screen time restriction, bedtime relaxation, sleep induction facilitated by generative AI, and technology-facilitated transition from sleep to wakefulness. Mapping to the stage-based model of personal informatics systems (37), we found that the design ideas cover all four stages of a self-tracking process, notably the action stage. Prior studies of consumer sleep health technologies have placed undue emphasis on sense-making of the rich flux of data collected with these technologies, centered on the data collection and reflection stages of self-tracking (14, 17, 13). To engage university students, however, sleep health technologies need to provide behavioral and psychological interventions that guide users to take actions within their lifestyle context. Prior cohort studies in sleep science have established initial evidence of behavioral intervention, such as cognitive behavior therapy for insomnia (i-CBT), in improving the sleep quality of university students (29, 62, 64, 63). Sleep health technologies have great compatibility with those intervention protocols but need to expand their value proposition for university students. Our analysis of the design ideas generated two major new findings. First, instead of considering sleep health technologies as stand-alone gadgets, our participants contextualized them as a part of a larger personal technology ecosystem and frequently integrated other technologies such as IoT, VR, and ChatGPT into their proposed app features. Another new finding is that university students demand sleep technologies to benefit their study and learning other than sleep health itself. They want to learn new skills, improve learning retention, or build investment portfolios while improving their sleep hygiene. On top of that, sleep health technologies targeting university students need to be entertaining, fun, and creative, as indicated by the design ideas under the themes of gamification. Collectively, those features may contribute to long-term user engagement because of their potential accumulative benefits appealing to the target population other than sleep health itself.

The present study has several limitations. First, our participants are predominantly male and mostly technology savvy. Second, the methodology of co-design needs to be used with caution when applied to the digital health context. It is important to note that the ideas generated through co-design with layman users may not always be clinically sound, and additional attention should be placed on issues such as privacy, data ownership and ethics. Third, some of the ideas, such as cash penalty, despite being innovative, are hard to implement. As such, the ideas generated through the co-design workshops will require post-filtering to derive a list of implementable and ethically sound features.

## 5 Conclusions

We have presented the insights and design ideas of sleep health technologies generated through three co-design workshops with university students. The ideas align well with the stage-based self-tracking model and cover all four stages, from preparation to action. Notably, new ideas surfaced under the themes of sleep environment optimization, online community, gamification, generative AI, materializing sleep with learning, and personalization. In our next step, we will prototype new sleep health technologies based on the ideas generated in the co-design process to promote sleep health among university students.

## Data Availability

The original contributions presented in the study are included in the article/Supplementary Material, further inquiries can be directed to the corresponding author.
